# A novel microfluidic wound model for testing antimicrobial agents against *Staphylococcus pseudintermedius* biofilms

**DOI:** 10.1186/1477-3155-12-1

**Published:** 2014-01-13

**Authors:** Jacob Terry, Suresh Neethirajan

**Affiliations:** 1 BioNano Laboratory, School of Engineering, University of Guelph, Guelph, ON N1G 2 W1, Canada

**Keywords:** Microfluidics, Biofilms, *Staphylococcus pseudintermedius*, Wound model, Antibiofilm

## Abstract

**Background:**

Current methods for testing treatments for veterinary surgical site infections can successfully emulate elements of a chronic wound, but these are time consuming and costly, requiring specialized laboratory equipment and considerable space to house study animals. Microfluidic devices however, can be coated with collagen and maintained at basal body temperature, providing a more cost-effective and space-saving model of a chronic wound. Our study assesses the applicability of a new microfluidic model by testing the activity of DispersinB against biofilms of methicillin-resistant *Staphylococcus pseudintermedius* (MRSP); DispersinB has been shown to prevent biofilm growth of *Staphylococcus epidermidis*, another prominent wound colonizer.

**Results:**

We successfully developed a microfluidic model to examine the effects of antimicrobial therapy on biofilms formed by organisms associated with wound infections in companion animals (e.g. MRSP). Although, we were unable to recapitulate previous findings that DispersinB-Gentamycin is highly effective against Staphylococcal biofilms using this model, we were able to confirm its effect in a microtitre plate. Differences in the experimental conditions likely account for this result (e.g. strains tested, flow conditions, treatment time, etc.). In the microtitre plate assay, DispersinB inhibited biofilm growth after a 24 hour period; there was an inverse relationship between the concentration of DispersinB-Gentamycin and the amount of biofilm remaining following treatment. Collagen-coated microtitre plates showed a similar result, but this did not correlate as well; collagen, the most abundant protein in the body may help to retain the biomass of treated biofilms.

**Conclusions:**

Our model may be useful in examining the effect of treatment on wound infections, although we acknowledge that in this model the test organisms may be more recalcitrant to antimicrobials than in other published systems. We contend that this may in fact better represent the conditions in vivo, where organisms associated with chronic wound infections are highly resistant to antimicrobials.

## Introduction

Methicillin-resistant *Staphylococcus pseudintermedius* (MRSP) bacterial biofilms have rapidly emerged as a serious complication in surgical site and wound infections in companion animals [1]. MRSP infections are being reported with increasing frequency in veterinary hospitals and have become the leading cause of pyoderma and surgical site infections in dogs [1,2]. MRSP infections are of tremendous concern in companion animals because they are challenging to eradicate, as they are typically recalcitrant to traditional antimicrobial therapy [1-3]. Therefore, it is imperative that the foundations of antimicrobial resistance be better understood.

Antimicrobial treatments may be tested in chronic animal wound infections on living animals, but this can lead to undesired consequences if the treatment were to have adverse effects, which may actually worsen infection or the presentation of the wound [1-4]. One way to avoid in vivo studies in veterinary hospitals would be to simulate a wound using synthetic material where possible [5]. Ideally, such models would display all the characteristics of an animal wound without having to use the animal as a test subject [2]. Some studies have been conducted that look at this possibility [6-8], but they primarily use materials to simulate the environment without necessarily simulating the wound itself. Finding a method that increases the accuracy of replicating wound tissue, which at the same time is inexpensive and saves both space and materials, would be most desirable. Such a model could emulate a true wound environment, while remaining easily deployable and could be repeated a sufficient number of times to facilitate advanced statistical analysis and model prediction.

Microfluidic technology is a relatively new field that can be applied to studying, evaluating, and understanding primary biofilm growth characteristics, ideally as a means to improve antimicrobial therapies that target pathogenic biofilms [9]. Microfluidic-based wound models can overcome the drawbacks associated with animal studies in testing antimicrobial agents, such as variability and lack of reproducibility. Microfluidics’ well-controlled reaction conditions, accurate delivery of drugs, and lower dead volumes will enable the development of novel skin models for testing antimicrobial agents against biofilms in a wound setting. Studies have shown that copious amounts of biofilm will readily form in channels of microscopic size [10], which can allow researchers to study biofilm growth in a confined, controllable, and reproducible space. For example, models with small channel volumes and high surface areas can be used to mimic the spatial environment of a blood vessel [11]. Microfluidic technology can be applied to more precisely understand the evolution of antimicrobial resistance in biofilms by creating dynamic concentration gradients, such as those found at sites where biofilms form [12,13]. Microfluidics can also be used to study the spatial and temporal growth of microorganisms, as well as motility and chemotaxis [12,13]. In this study we investigated the efficacy of an antimicrobial agent against MRSP biofilms in a wound model we developed using microfluidic platforms.

DispersinB, a glycoside hydrolase enzyme, has been shown to successfully inhibit biofilm growth in *Staphylococcus* species, specifically *S. epidermidis*[14-16]. This suggests that DispersinB may be an effective candidate for testing the viability of new wound infection models; these results can then be verified using established microtitre plate assay methods [15,17]. The efficacy of DispersinB has been investigated using conventional techniques for studying wound-associated bacteria, such as *Klebsiella penumoniae* and *Staphylococcus epidermidis*[18]. However, its effect on MRSP, another key wound infection-causing bacterium, has not been investigated. At wound sites, a range of multidrug resistant pathogens can be encountered, resulting in tremendous morbidity, mortality, and healthcare costs for both animals and humans. Biofilm formation has been hypothesized as the reason for the emergence of highly resistant and pathogenic MRSP clones [19].

Therefore, we examined the antimicrobial resistance profiles of MRSP biofilms by evaluating the efficacy of DispersinB in combination with Gentamycin in our newly developed model. Collagen coated polymeric microfluidic platforms, coupled with microscopy, enabled the in situ observation of antimicrobial susceptibility and the evolution of distinct microbial morphologies over the course of biofilm formation. Our results helped to determine an effective minimal concentration of DispersinB and Gentamycin that may be sufficient to eradicate MRSP biofilms. Ultimately, this microfluidic assay may be used to improve the clinical management of wound associated biofilm infections in companion animals.

## Materials and methods

### Bacterial strains and antimicrobial agent

The study bacteria chosen for use in this investigation were selected based on data from a previous study of clarithromycin efficacy against MRSP biofilms [1]. The three strains chosen were selected to demonstrate efficacy of DispersinB with gentamicin against bacteria of varying adherence capabilities. High, medium, and low adherence capability were represented by the A12, A92, and SP102 isolates, respectively. The mixture of DispersinB and gentamicin used was 1 mg/mL gentamicin and 200 μg/mL DispersinB, which was suspended in 50 mM sodium phosphate (pH 5.8) and 100 mM NaCl (Kane Biotech Inc., Winnipeg, Canada).

### Culture and dye methods

Each strain was sub-cultured from frozen stock onto Columbia agar plates with 5% sheep blood and grown for 24 hours at 35°C. For microfluidic tests, test tubes containing 5 ml tryptic soy broth with 1% glucose (TSB-G) were inoculated with A92 isolates to the equivalent turbidity of a 0.5 McFarland standard, then incubated for 3 hours at 35°C and diluted with 5 ml of sterile water. Six micro centrifuge tubes were filled with 1 ml of the diluted solution and centrifuged for 3 minutes at 12,000 rpm. The supernatant was removed and discarded, and the cells were suspended in an additional 1 ml of water, and then centrifuged again. This washing process was repeated a total of three times. To make the dye solution, 3 μL of dimethyl sulfoxide with 1.67 mM SYTO 9 dye and 1.67 mM propidium iodide was suspended in 1 ml water. Two hundred μL of this dye solution was added to the washed cells (currently suspended in 1 ml water) to yield 1.2 ml of dyed cell solution. Tubes were then stored in the dark at room temperature for 30 minutes before use.

### Preparation of microfluidic devices

Devices used in these experiments were a simple Y-channel device with 3-in-1 units, with a 3 mm wide main channel, 1 mm tall inlet and outlet channels, and a channel height of 400 μm, providing a total channel volume of 60 μl. The devices used for the final experiments were coated with 75 μg/ml rat-tail collagen, type I in 0.05 M hydrochloric acid by filling through the outlet channel. After 2 h, the device was aspirated, washed with sterile water, and left to dry at room temperature for 1 h before use.

### Bacterial flow and incubation

**Figure 1:**
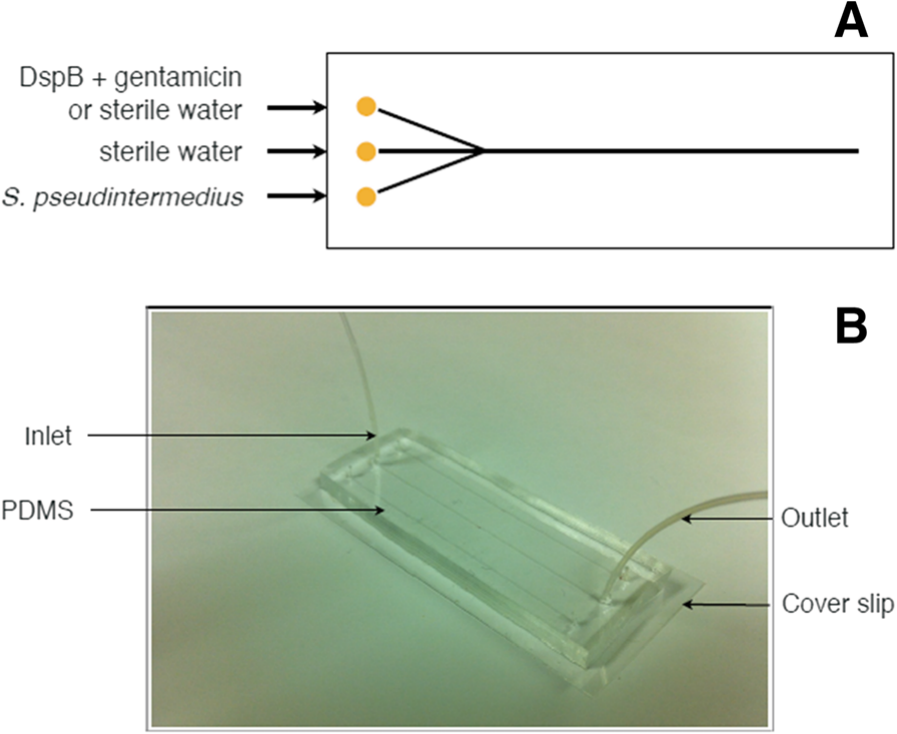
**Schematic diagram (A) of the microfluidic wound model experimental setup.** A Y-shaped microfluidic platform with a microchannel served as a cell culture well mimicking an animal wound. This 450 μm wide, 125 μm deep channel had a reservoir volume of 30 μL, and was connected on each side by two inlets and one outlet. Single channel microfluidic device **(B)** formed from PDMS using soft lithography techniques. For collagen-coated experiments, collagen was flown through the inlet before testing the antimicrobials.

Five hundred μL of the dyed bacterial solution was added to a glass syringe. The device was mounted onto a microscope, and the syringe was connected via tubing with Luer tapers. An outlet tube was connected to collect any extra liquid. Using a syringe pump, bacteria were flowed at a rate of 200 μl/h through the middle channel for 1 h, while the top and bottom inlet reservoirs were sealed using Luer-compatible syringes or seals (Figure 1A). Images were taken of the bacteria flow in the outlet channel every minute during the bacterial flow process. Tubing was disconnected and the device was incubated for 4, 8, 12 or 24 hours at 35°C. Refer to Figure 1B for an illustration of the operation of our microfluidic device.

### Efficacy testing of dispersin B and/or sterile water

After the device was incubated, 500 μl of sterile water was added to a glass syringe and 500 μl of either DispersinB or sterile water was added to a second glass syringe. The device was mounted onto a microscope and the syringes were connected via tubing with Luer tapers. An outlet tube was connected with a micro-centrifuge tube to collect the final solution for enumeration testing. Using a syringe pump, DispersinB or sterile water was flowed at a rate of 100 μL/h through the top channel, and sterile water was flowed at a rate of 100 μl/h through the bottom channel for 4 hours, while the middle inlet reservoir was sealed using a Luer-compatible syringe or seal (Figure 1B). Images were taken every minute for the first 2 h at the same location in the outlet channel that was used.

### Enumeration testing

Micro centrifuge tubes were used to collect liquid suspensions of cells from the outlet tube; serial dilutions of these solutions were made to enumerate the collected cells. We plated the 1:10, 1:100, and 1:1,000 dilutions onto Columbia agar with 5% sheep blood. The plates were incubated at 35°C for 24 h before the colonies were counted.

### Image analysis

Bacterial flow and post-incubation images were numbered in sequence prior to image analysis. Using MATLAB Version R2013B (Mathworks Inc., Natick, MA), images were read in order and Otsu thresholding was performed to determine fluorescent intensity and surface coverage. To numerically justify the results from the image analysis readings, the area under the curve formed in all surface coverage and intensity graphs was measured and compared.

### Micro-titre plate assay

Epidemiologically unrelated MRSP isolates from dogs from different geographic regions were screened for biofilm production via microtiter plate assay (MPA). Briefly, overnight cultures were suspended in 5 ml of tryptic soy broth (TSB) supplemented with 1% glucose to achieve a turbidity equivalent to a 0.5 McFarland standard (~10^8^ CFU/ml). 200 μl of each inoculum was transferred in triplicate to a 96-well polystyrene microtiter plate and incubated under aerobic conditions for 24 h at 35°C. Following incubation, the plates were washed three times with phosphate buffered saline (PBS) to remove non-adherent cells and then heat fixed at 60°C for 60 minutes. Adhered cells were dyed with 0.1% (w/v) of crystal violet for 15 minutes and air dried at room temperature. After resolubilization with 95% ethanol, optical density (OD) reading of each well of the microtiter plate was assessed, taken at 570 nm (OD570). Readings of replicates for each isolate were averaged and subtracted from the OD570 reading of the negative control. OD570 was used as indication of biofilm production. Isolates were classified as biofilm producers if OD570 was >0.200 and further classified as strong, moderate, weak, or zero biofilm formers based on their final OD570 reading.

## Results and discussion

**Figure 2:**
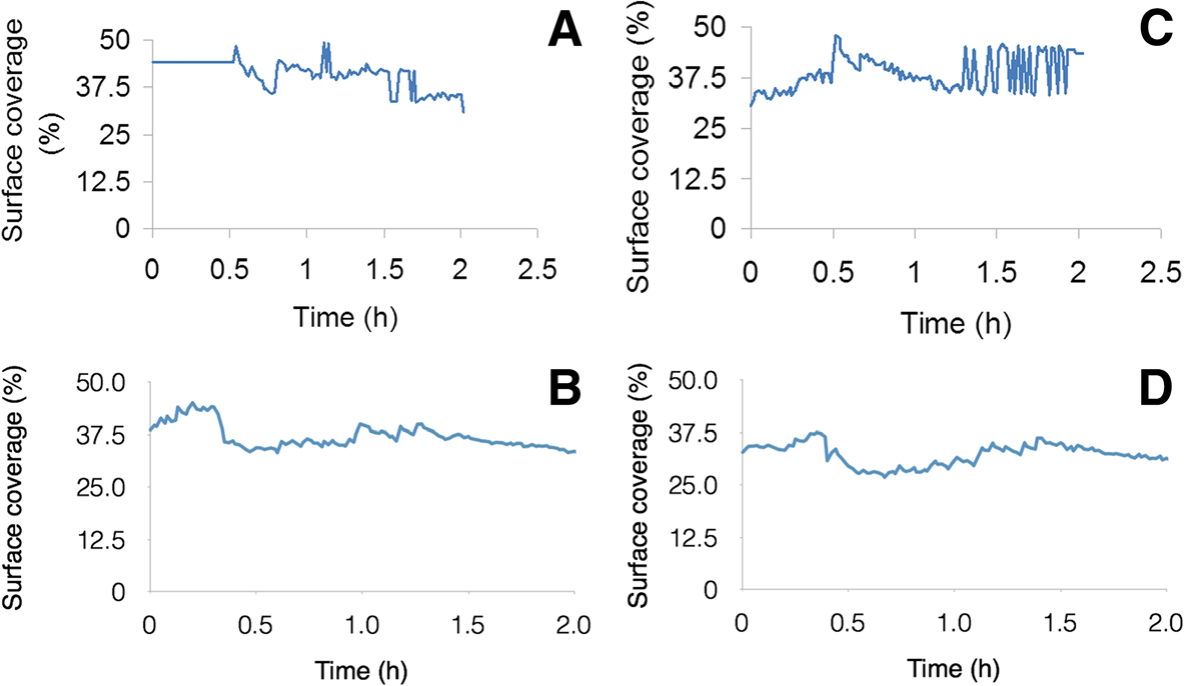
**Biofilm coverage inside the microfluidic channels.** This figure demonstrates the biofilm surface coverage during a period of 2 hours after each of the denoted time points were reached, using water and DispersinB treatments. Panels are as follows: **(A)** 24 hours with DispersinB and water, **(B)** 24 hours with water, **(C)** 12 hours with DispersinB and water and **(D)** 12 hours with water.

We monitored biofilm coverage inside the microfluidic device after 4, 8, 12, and 24 h of biofilm growth. We began monitoring at each of these time points and performed constant monitoring (images taken every minute) over a 2 h period. When the percent surface area was calculated and the readings were graphed, similar patterns in the structure of the graphs were observed. We focused on the results obtained from 12 and 24 h biofilms, as these time points should yield mature structures consistent with chronic wound infections. Surface coverage readings for all four time points showed that 25-50% of the surface was consistently covered with biofilm (Figure 2A, 2B, 2C and 2D) and that relatively stable levels of coverage were maintained over the 2 h imaging period.

The intensity of biofilm growth in regions across the channel was parabolic as expected, since bacteria flowed through the middle channel before incubation. Unexpectedly, when DispersinB-Gentamycin was used instead of water in the top channel, it was found that the biofilm intensity was greater on average. The average normalized intensity value for 24 h biofilms was 0.536 for DispersinB-Gentamycin and 0.500 for water alone, while at 12 h biofilms it was found to be 0.599 for DispersinB-Gentamycin and 0.411 for water alone. We propose that the apparent increase in biomass may be a response to the stress induced by treatment with antimicrobials. It is not uncommon for the biofilm architecture to be altered in the presence of inimical forces. For example, shear forces applied to biofilm have been shown to result in an increase biofilm density [20].

**Figure 3:**
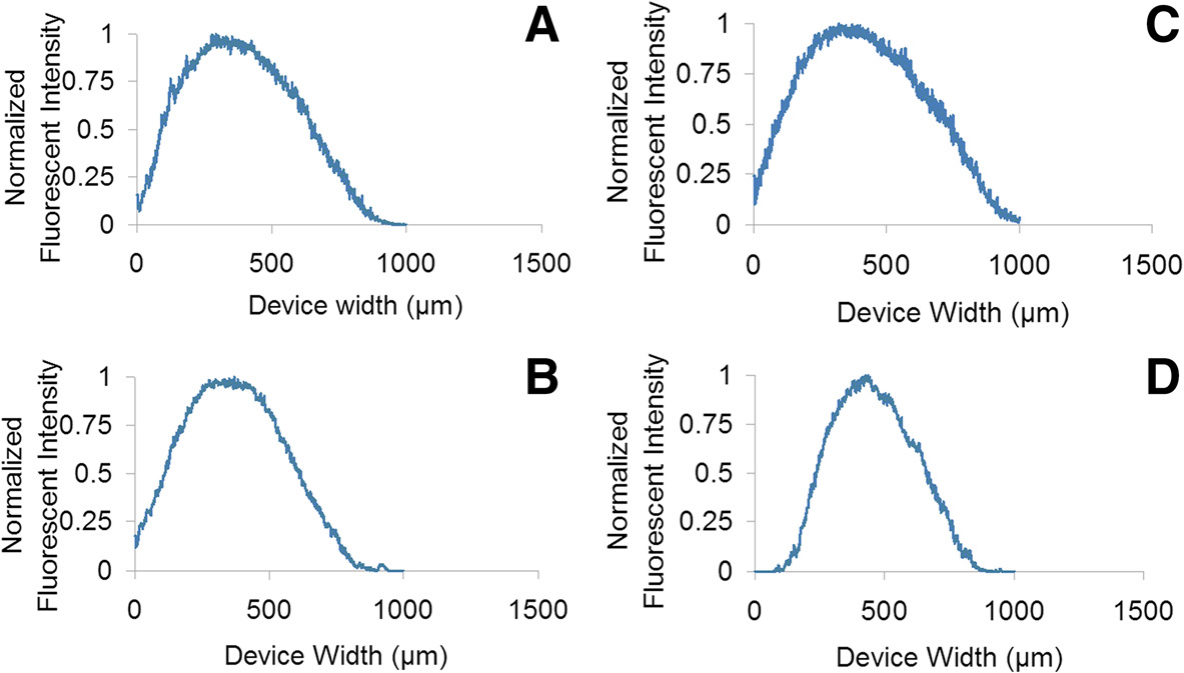
**Biofilm fluorescence intensity inside the channels.** This figure demonstrates the average fluorescence intensity (normalized) in the channels during a period of 2 hours after the biofilms had reached the desired time point. Images are as follows: **(A)** 24 hours with DispersinB **(B)** 24 hours with water, **(C)** 12 hours with DispersinB, and **(D)** 12 hours with water.

Comparing the top half of the channel (Figure 3A) with the bottom half of the channel (Figure 3B), we noted that there was a great deal of contrast between these images. All of our experiments produced data with a bias towards the top half of the channel (more biomass is located in this area), indicating that the design of the microfluidic device or the biofilm itself may favour growth on that side of the device. After 24 h, the top half of the channel held an average normalized value of 0.747 when DispersinB-Gentamycin (Figure 3A) or water alone (Figure 3B) was used, while the bottom half held an average normalized value of 0.324 when DispersinB was used and 0.253 when water alone (Figure 3B and 3D) was used. After 12 h, the top half of the channel held an average normalized value of 0.765 when DispersinB-Gentamycin was used (Figure 3C) and 0.499 when water was used, while the bottom half held an average normalized value of 0.434 when DispersinB-Gentamycin was used and 0.322 when water alone was used. These results indicated that there was a greater accumulation of biofilm in the top channel. Again, the accumulation (or lack of dispersion) was enhanced when the biofilms were treated with DispersinB-Gentamycin, which as suggested earlier may be due to a biofilm-promoting response to treatment.

**Figure 4:**
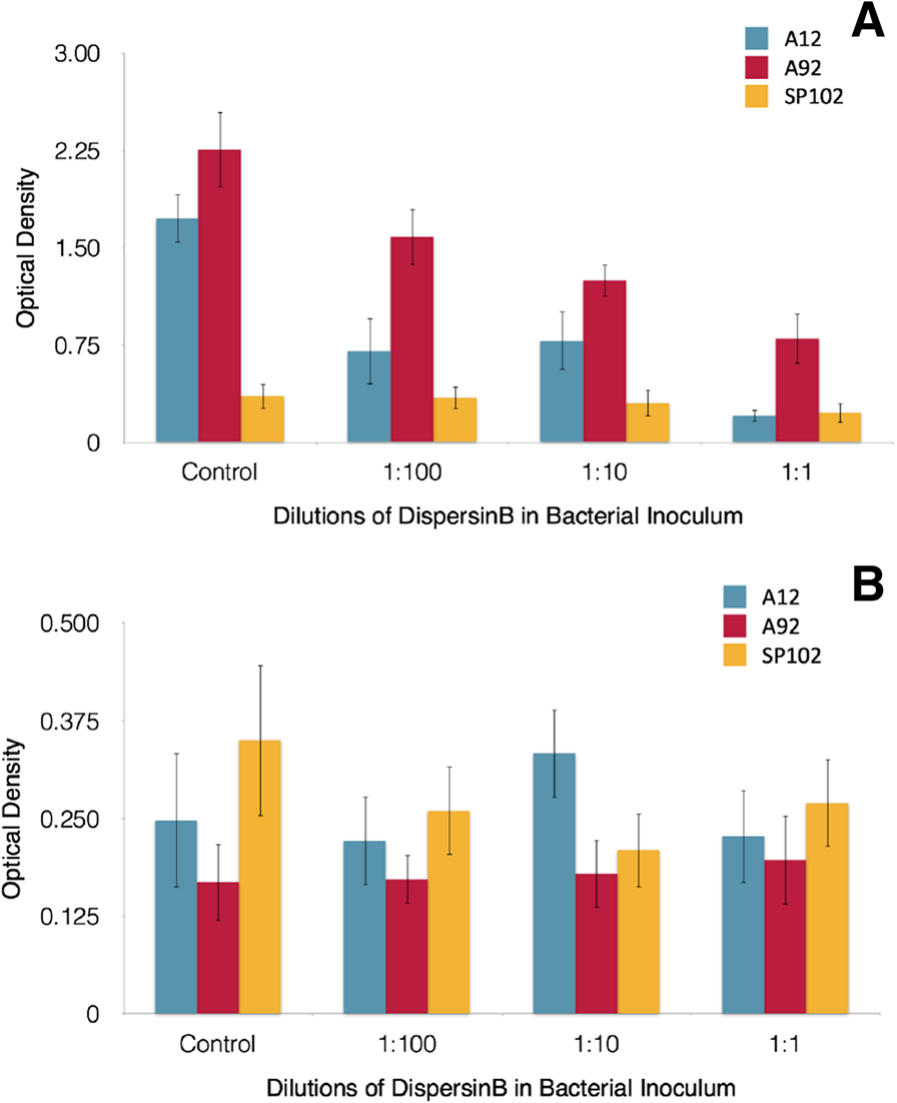
**Microtitre plate assay findings.** This figure demonstrates the attachment of MRSP biofilm strains to **(A)** uncoated, and **(B)** collagen-coated polystyrene surfaces after 24 h using various concentrations of DispersinB-Gentamycin as noted. Crystal violet was used to determine optical density.

Microtitre plate assay readings confirmed previous reports showing that DispersinB may be effective against wound infection biofilms. We determined that there was an inverse relationship between the DispersinB-Gentamycin concentration and the level of biofilm growth in uncoated wells (Figure 4A). There was also a relationship between the effectiveness of DispersinB-Gentamycin and the biofilm forming capabilities of the selected strain; better biofilm forming strains showed a greater percent reduction in the biomass following treatment. MRSP strain A12 was reduced to 40.8% of its control, while A92 was reduced to 70.1%, and SP102 was only slightly reduced to 96.1% using a 1:100 dilution of DispersinB-Gentamycin. This decline was more prominent using1:1 dilutions of DispersinB-Gentamycin; A12 was reduced to 11.9% of its control value, A92 was reduced to 35.5%, and SP102 was reduced to 63.6%. For collagen-coated plates (Figure 4B), the results showed a weaker correlation. Taking any error into account, each isolate showed a relatively stable level of biofilm density when comparing control values to all the concentrations of DispersinB-Gentamycin tested. The optical density values were much lower in collagen-coated tests (Figure 4B), with values routinely less than 0.5 compared to values that ranged up to an optical density of 2.258 in uncoated tests. Rat tail collagen was used for these studies, which is economical and readily available, but we acknowledge that this may not be the ideal substrate for MRSP attachment. In the future, we could test more relevant sources of collagen to determine if this improves overall biofilm formation.

## Conclusions

Microfluidic tests performed as described in this study showed that there was minimal biofilm shearing from our microfluidic device, even following long periods of idle incubation. Although, we determined that the ability of DispersinB-Gentamycin to remove biofilm growth was not dramatically different than treatment with water, we contend that we have developed a successful model to study chronic wound infections in animals. Uncoated microtitre plate assays did confirm that DispersinB-Gentamycin is capable of effectively removing biofilm growth, which appears to be particularly effective against strains that are strong biofilm formers. Additional studies with our microfluidic device may be necessary to determine the best conditions for establishing a biofilm that resembles those that form during wound infection. We would suggest focusing on a strong biofilm forming strain, such as A12, possibly incorporating smaller channel widths or other sources or preparations of collagen. Wound infections are typically polymicrobial [21]. Hence co-culture studies may more accurately reflect the conditions present in vivo. We also would consider incorporating host cells into our model, such as endothelial cells. Ultimately, these results are preliminary, but we have developed a base model that may be modified to better emulate the conditions experienced in vivo during a chronic wound infection. Our model is easy to use, space-saving, and economical. Such a model may be highly effective in mimicking a wound infection without resorting to invasive testing of antimicrobial therapies in animals with wound and surgical site infections.

## Competing interests

The authors declare that they have no competing interests.

## Author’s contributions

JT and SN designed the project. JT performed the experiments and wrote the manuscript with SN. SN contributed to the conceptual design and manuscript preparation. Both authors read and approved the manuscript.
